# Facteurs prédictifs de fistule anastomotique après colectomie pour cancer

**DOI:** 10.11604/pamj.2022.42.129.33570

**Published:** 2022-06-16

**Authors:** Amine Zouari, Abderrahmen Masmoudi, Fatma Khanfir, Salma Ketata, Haithem Rejab, Ahmed Bouzid, Issam Loukil, Imen Zribi, Skander Talbi, Amine Abdelhedi, Bassem Abid, Salah Boujelben

**Affiliations:** 1 Université de Sfax, Faculté de Médecine de Sfax, Centre Hospitalo-Universitaire Habib Bourguiba Sfax, Service de Chirurgie Générale, Sfax, Tunisie,; 2 Université de Sfax, Faculté de Médecine de Sfax, Centre Hospitalo-Universitaire Hédi Chaker Sfax, Service de Gynécologie Obstétrique, Sfax, Tunisie,; 3 Université de Sfax, Faculté de Médecine de Sfax, Centre Hospitalo-Universitaire Habib Bourguiba Sfax, Service d'Anesthésie Réanimation, Sfax, Tunisie,; 4 Service de Chirurgie Générale Tataouine, Tataouine, Tunisie

**Keywords:** Tumeur colon, chirurgie colorectale, fistule anastomotique, morbidité, Colon cancer, colorectal surgery, anastomotic fistula, morbidity

## Abstract

**Introduction:**

en chirurgie du cancer colique, la fistule anastomotique (FA) est la complication la plus redoutée. Le but de cette étude était de déterminer les facteurs prédictifs de fistule anastomotique après résection pour cancer colique ainsi de décrire l´impact de cette complication sur la mortalité et la durée du séjour postopératoire.

**Méthodes:**

nous avons mené une étude retrospective, descriptive et analytique, allant du 1^er^ janvier 2013 au 31 d écembre 2020, dans le service de chirurgie générale de l´Hôpital Habib Bourguiba de Sfax, Tunisie.

**Résultats:**

nous avons colligé 163 malades opérés pour cancer colique. L´âge moyen était de 62,7 ans avec un sexe ratio de 1,36. Les suites opératoires étaient simples dans 64,4% des cas et compliquées dans 35,6%. La morbidité chirurgicale était essentiellement représentée par les fistules anastomotiques, identifiées chez 22 malades. Au terme de notre travail, il ressort que les facteurs prédictifs de la survenue de cette complication sont: le diabète avec un p = 0,04, le tabagisme avec un p = 0,01, l´hypoalbuminémie avec un p = 0,01, l´hémoglobine préopératoire inférieure à 10g/dl avec un p < 0,01, la localisation au niveau de l´angle colique gauche avec un p = 0,02, la transfusion peropératoire avec un p <0,01 et une durée opératoire supérieure à 180 min avec un p = 0,04. Par ailleurs, la survenue de FA était accompagnée d´un taux de mortalité spécifique de 9% et avait prolongé de façon significative la durée de séjour postopératoire.

**Conclusion:**

la prévention de la FA doit s´inscrire dans le cadre d´une prise en charge multimodale du patient avec essentiellement un apport nutritionnel et une correction d´une éventuelle anémie en préopératoire.

## Abstract (en)

**Introduction:**

in colon cancer surgery, anastomotic fistula (AF) is considered the most feared complication. The purpose of this study was to identify predictive factors associated with anastomotic fistula after colon cancer surgical resection and to describe the impact of this complication on mortality and postoperative length of stay.

**Methods:**

we conducted a retrospective, descriptive and analytical study in the Department of General Surgery at the Habib Bourguiba Hospital in Sfax, Tunisia from 1^st^ January 2013 to 31 December 2020.

**Results:**

we collected data from the medical records of 163 patients who had undergone surgery for colon cancer. The average age of patients was 62.7 years with a sex ratio of 1.36. The postoperative course was uneventful in 64.4% of cases and complicated in 35.6% of cases. Surgical morbidity was mainly due to anastomotic fistulas (22 patients). This study demonstrated that predictors of the development of this complication were: diabetes p = 0.04, smoking p = 0.01, hypoalbuminaemia p = 0.01, preoperative haemoglobin less than 10g/dl, p < 0.01, anastomotic fistula located in the left colonic angle p = 0.02, perioperative transfusion p <0.01, and duration of surgery longer than 180 min p = 0.04. Moreover, the occurrence of anastomotic fistula was associated with specific mortality rate (9%) and significantly prolonged postoperative length of stay.

**Conclusion:**

the prevention of anastomotic fistulas should be part of a multimodal approach based on the correction of nutritional deficiencies and possible pre-operative anemia.

## Introduction

Le cancer du côlon représente un problème majeur de santé publique. C´est un cancer fréquent avec un sexe ratio de 1,1. Il représente avec celui du rectum la 3^e^ cause de décès par cancer tous sexes confondus [1]. En chirurgie du cancer colique, la fistule anastomotique (FA) reste la complication la plus redoutée. Elle est responsable d'une augmentation de la morbi-mortalité et de la durée d'hospitalisation [2]. La grande majorité des publications étudiant les facteurs de risque de fistule anastomotique ont inclus à la fois les résections coliques et rectales. Toutefois, la FA est généralement plus fréquente après résection rectale, d´où le risque d´un biais de sélection. Par conséquent, les facteurs de risque spécifiques à la chirurgie colique restent dissimulés. Actuellement, plusieurs auteurs recommandent de considérer le cancer du côlon et celui du rectum comme des entités tumorales différentes [3,4]. En fait, une meilleure connaissance des facteurs prédictifs spécifiques de cette complication permet d´orienter et d´améliorer la prise en charge. Dans cet esprit, nous avons mené ce travail pour analyser les facteurs prédictifs de fistule anastomotique après résection pour cancer colique.

## Méthodes

**Cadre de l'étude**: cette étude s'est déroulée au Service de chirurgie générale de l´Hôpital Habib Bourguiba de Sfax, Tunisie. Il s´agit du plus grand centre hospitalo-universitaire de la région du sud du pays.

**Type d´étude**: nous avons mené une enquête observationnelle rétrospective de type descriptive et analytique allant du 1^er^ janvier 2013 au 31 décembre 2020, portant sur des patients opérés d´un cancer colique.

**Conception de l´étude**: notre travail se distingue par l´attention portée à la chirurgie du cancer colique uniquement. En effet, la majorité des études publiées incluent à la fois les résections coliques et rectales. Par conséquent, les facteurs de risque spécifiques à la chirurgie colique restent dissimulés. Dans cet esprit, et pour minimiser les biais, nous avons étudié spécifiquement les résultats de la chirurgie colique avec rétablissement immédiat de la continuité.

**Participants à l'étude**: l'étude a inclus tous les patients ayant eu un rétablissement immédiat de la continuité digestive après chirurgie élective ou urgente pour cancer colique dans notre service. Les tumeurs de la charnière recto-sigmoïdienne, les anastomoses sous-Douglasiennes et les dossiers incomplets n´ont pas été inclus. Durant la période de l´étude, nous avions opéré 329 patients d´une tumeur colique. Après application des critères d´inclusions et d´exclusion, 163 patients étaient inclus dans notre étude.

**Variables**: nous avons étudié: l´âge, le sexe des patients, les antécédents médicaux et/ou chirurgicaux, les paramètres biologiques préopératoires, les constations per-opératoires, les caractéristiques anatomopathologiques, la durée d´hospitalisation les suites opératoires ainsi que la survenue de fistule anastomotique en postopératoire.

**Définitions**: nous avons pris en compte la définition large de la FA comme recommandé par la conférence de consensus de 2020 publié dans « World Journal of Surgery » [5]. De ce fait, la FA était définie par la présence d´au moins un des critères suivants: 1) présence de pus ou de contenu entérique dans le liquide de drainage; 2) présence de collection abdominale ou pelvienne en regard du site de l´anastomose à l´imagerie; 3) suite de produit de contraste en péri-anastomotique au scanner; 4) déhiscence de l´anastomose découverte lors d´une réintervention pour péritonite postopératoire.

**Analyse des données**: la saisie et l´exploration des résultats étaient réalisées par le logiciel SPSS (*Statistical Package for the Social Sciences*) dans sa version standard 20. Pour les variables qualitatives nous avions utilisé le test Khi-deux. Le test exact de Fisher était utilisé pour les faibles effectifs. Pour les variables quantitatives à distribution normale, nous avions utilisé le test de Student. Dans le cas contraire, nous avions utilisé le test de Mann-Whitney après échec des tentatives de transformation des variables. La différence était jugée significative quand le coefficient de corrélation (p) était inférieur à 0,05. Dans une étape suivante, nous avions mené une analyse multivariée en utilisant des variables indépendantes ayant un p ≤ 0,2 par le biais de modèles de régression logistique. A l´issue de ces analyses, des courbes ROC (*Receiver Operating Characteristic*) étaient réalisés afin de déterminer un seuil prédictif de fistule anastomotique.

**Considération éthique**: afin de garantir la confidentialité des informations personnelles des patients, les données ont été recueillies sur des fiches d'enquête anonymisées tout au long de cette enquête.

## Résultats

**Figure 1:**
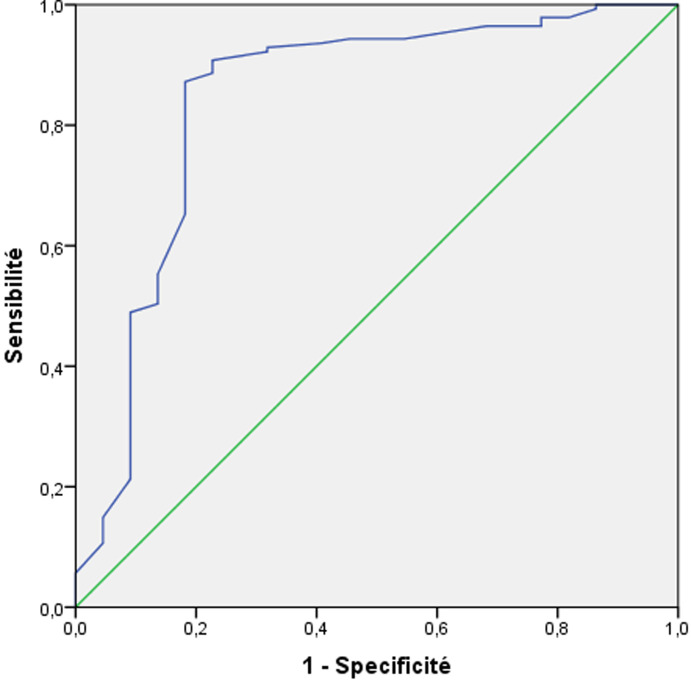
courbe ROC de l’hémoglobine préopératoire

Cent soixante-trois patients ont été inclus dans l´étude (Figure 1). L´âge moyen au moment de l´intervention était de 62,7 ans avec un sexe ratio de 1.36. Les caractéristiques épidémiologiques, cliniques et paracliniques sont représentées dans le Tableau 1. Le taux de morbidité globale était de 35,6%. La morbidité chirurgicale était essentiellement représentée par les fistules anastomotiques identifiées chez 22 malades (13,5%). Le délai moyen du diagnostic de cette complication était de 7 jours avec des extrêmes de 3 à 15 jours. La fistule était bien dirigée dans 7 cas, associée à une collection profonde dans 9 cas et associée à une péritonite postopératoire dans 6 cas. La comparaison des principaux éléments cliniques, paracliniques, et évolutifs entre les patients qui ont présenté une FA post-opératoire et ceux qui n'ont pas présenté cette complication, figure sur le Tableau 2. En analyse bivariée, il ressort au terme de notre travail que les facteurs prédictifs de la survenue de cette complication sont: le diabète avec un p = 0,04, le tabagisme avec un p = 0,01, l´hypoalbuminémie avec un p = 0,01, l´anémie préopératoire avec un p < 0,01, la localisation au niveau de l´angle colique gauche avec un p = 0,02, la transfusion peropératoire avec un p < 0,01, ainsi que la durée opératoire avec un p = 0,04.

**Tableau 1:** caractéristiques de la population étudiée

Caractéristiques	Valeurs
Age	62,7 (±14,5)
**Sexe**	
Hommes	94 (57,7%)
Femmes	69 (42,3%)
**ASA**	
ASAI	111 (68,1%)
ASAII	44 (27%)
ASAIII	8 (4,9%)
**Antécédant et habitudes**	
Tabagisme	58 (35,6%)
HTA	42 (25,8%)
Diabète	33 (20,2 %)
Cardiopathie ischémique	19 (11,7%)
BPCO	5 (3,1%)
**Caractéristiques biologiques**	
Hg préopératoire	11,2 (±2)
Albuminémie préopératoire (g/L)	34,6 (±4,6)
**Voie d´abord**	
Laparotomie	126 (77,3%)
Laparoscopie	37 (22,7%)
**Localisation tumorale**	
Colon droit	61 (37,5%)
Colon gauche	102 (62,5%)
**Circonstances d´intervention**	
A froid	132 (81%)
En urgence	31 (19%)
Durée opératoire (min)	171 [45-400]
**Suites Opératoires**	
Morbidité Globale	58 (35,6%)
FA	22 (13,5%)
**Stade Tumoral**	
Stade I	15 (9,5%)
Stade II	57 (36,1%)
Stade III	56 (35,6%)
Stade IV	28 (18,8%)
Mortalité postopératoire	7 (4,3%)
Durée de séjour postopératoire (jours)	7 [4-60]

Les variables quantitatives sont exprimées en moyenne ± écart type quand la distribution est gaussienne ou en médiane (interquartile) si elle est non gaussienne. Les variables qualitatives sont exprimées en effectif (pourcentage). Hg: hémoglobine, FA: fistule anastomotique

**Tableau 2:** étude comparative entre les deux groupes

Etude bivariée			
Variables Etudiées	Groupe FA	Groupe pas de FA	p
**Diabète**			0,04
Oui	8	25	
Non	14	116	
**Tabagisme**			0,01
Oui	13	45	
Non	9	96	
**Hypoalbuminémie**			0,01
Oui	14	29	
Non	5	59	
**Hémoglobine préopératoire (moyenne en g/dl)**	9,2	11,5	<0,01
Tumeur de l´ACG**			0,02
Oui	5	17	
Non	8	133	
**Transfusion peropératoire**			<0,01
Oui	6	4	
Non	16	137	
**Durée opératoire (moyenne en min)**	213	165	0,04
**Etude multivariée**			
**Variables étudiées**	**P**	**Odds Ratio**	**IC**
**Hypoalbuminémie**	0,03	7,5	[1,2; 49]
**Hb < 10 g/dl en préopératoire**	0,01	6,6	[3,6; 42]

Les variables quantitatives sont exprimées en moyenne ± écart type et les variables qualitatives en effectif. ACG: Angle colique gauche, FA: Fistule Anastomotique, IC: Intervalle de confiance

**Figure 2:**
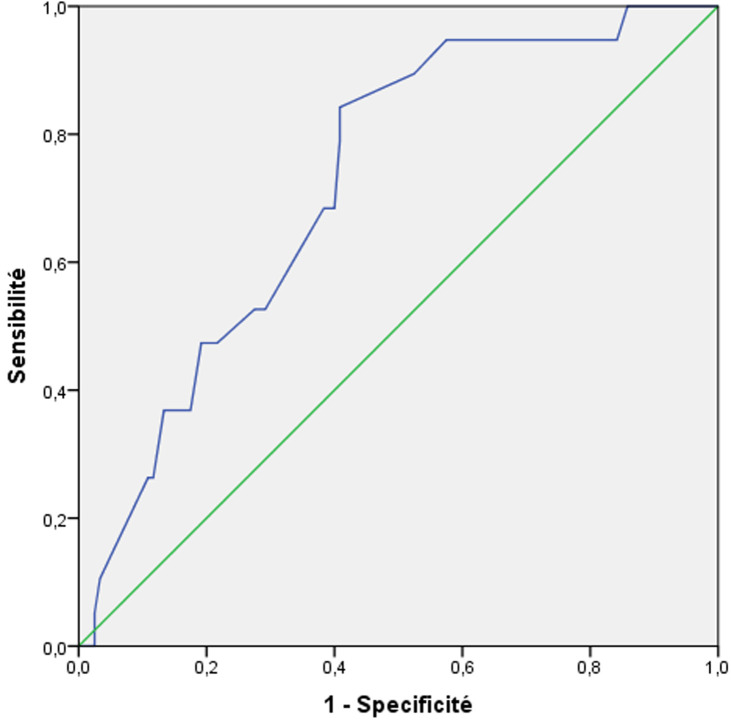
courbe ROC de la durée opératoire

L´analyse de la courbe ROC de l´hémoglobine préopératoire (Figure 1) montre une aire sous la courbe (ASC) de 0.84. Nous avions retenu la valeur de 10 g/dl comme « cut-off » avec une sensibilité de 82%, une spécificité de 81% et une valeur prédictive négative (VPN) de 96%. L´analyse de la courbe ROC de la durée opératoire montre une ASC de 0.72 (Figure 2). Nous avons retenu la valeur de 180 min comme « cut-off » avec une sensibilité de 52%, une spécificité de 71% et une VPN de 90%. En analyse multivariée, les facteurs prédictifs indépendants de fistule anastomotique sont l´hypoalbuminémie (OR= 7,5, IC à 95% 1,2; 49], p= 0,03) et hémoglobine préopératoire inférieure à 10g/dl (OR=6,6, IC à 95% [3,6; 42,9] p=0,01). Dans notre étude, nous avons remarqué une majoration du taux de mortalité postopératoire en présence de fistule anastomotique sans qu´il y ait une relation statistiquement significative avec cette complication (p = 0,24). En effet, ce taux passe de 4,3% à 9% en cas de présence de cette complication. Par ailleurs, la survenue de FA a prolongé de façon significative la durée de séjour postopératoire. Cette durée passe de 8,5 jours à 19,5 jours en cas de FA (p = 0,01).

## Discussion

La fistule anastomotique est la complication la plus redoutable de la chirurgie colique à l´origine d´une morbi-mortalité surajoutée. Le taux de FA après chirurgie pour cancer colique est hétérogène et varie de 2,7% à 15,9% [6-8]. Dans notre étude, ce taux a été de 13,5%. Cette incidence, relativement importante, peut être expliquée étant donné que nous avons pris en compte l´ensemble des fistules cliniques et radiologiques, comme recommandé par la conférence de consensus de 2020 [9]. Cela fait que la comparaison avec les différentes séries doit se faire avec prudence. L'évaluation des risques est un élément fondamental de la prise de décision en chirurgie. Cette évaluation guide la prise en charge ultérieurement. Elle peut même influencer la prise de décision en peropératoire, incitant le chirurgien à changer de stratégie devant une anastomose à risque. L´intérêt de notre étude réside dans l´identification de facteurs de risques de FA propres à la population de notre région. Ces résultats aideront à mieux identifier les patients à risque avant la chirurgie et ainsi à améliorer les résultats de la chirurgie colique.

Bien que plusieurs études ont identifié le diabète comme facteur de risque de FA [10-13], d´autre études suggèrent que le diabète n´augmente pas l´incidence de cette complication [14,15]. Toutefois, Ziegler *et al*. ont démontré qu´il augmente de façon significative la gravité de la FA (26,3% de mortalité versus 6% chez les non diabétiques) [15]. Dans notre série le diabète type 2 était identifié comme facteurs de risque de FA après chirurgie colique avec un p = 0,04. En outre, Baucom *et al*. [16] ont étudié spécifiquement l´effet du tabac sur les anastomoses après chirurgie colique sur 246 patients. Les fumeurs avaient 4 fois plus de risques de FA (OR: 4,2, IC [1,3; 13,5], p = 0,02). Étant donné qu'il n'a pas été démontré que l'arrêt du tabac à court terme réduisait les FA, les recommandations en chirurgie digestive préconisent un sevrage tabagique quatre à huit semaines avant le geste opératoire et tout au long de la période postopératoire précoce [17]. Dans notre étude, les patients tabagiques pourraient avoir un risque majoré de développer une FA en postopératoire (p = 0,01), ce qui est en accord avec les études sus décrites.

Par ailleurs, une bonne oxygénation tissulaire étant indispensable au processus de cicatrisation, l´anémie préopératoire était reconnue comme facteur de risque de FA dans plusieurs études [18-21]. La plupart des auteurs considèrent une Hb = 11 g/dl comme limite acceptable avant toute chirurgie colorectale [22]. L´essai prospectif Néerlandais « LekCheck » a aussi identifié l´anémie préopératoire comme facteur de risque important de FA avec un OR de 5, considérant la valeur de 9,7g/dl comme « cut-off » [21]. Dans notre série, l´anémie préopératoire était significativement associée à la survenue de FA avec un p =0,01, notre cut-off était de 10 g/dl comme « cut-off » avec une sensibilité de 82%, une spécificité de 81% et une VPN de 96%. Dans la large cohorte de Hu *et al*., incluant 42483 patients opérés pour cancer colorectal, les patients avec hypoalbuminémie ont présenté trois fois plus de risque de développer une FA (OR = 3, p < 0.001) [23]. Dans notre série, les résultats sont concordants avec cette étude. L´hypoalbuminémie était identifiée en analyse multivariée comme facteur de risque indépendant de survenu de FA (OR= 7,5, IC à 95% [1,2; 49], p = 0,03).

Par ailleurs, dans une revue systématique récente, Charalambides *et al*. avaient analysé 16 études et ont montré que les pertes sanguines supérieure à 200ml et les transfusions peropératoires sont tous deux des facteurs de risque indépendants de FA [24]. Dans notre étude, la transfusion peropératoire apparait aussi comme facteur de fistule anastomotique en étude bivariée avec un p < 0.001. Dans notre série, une durée opératoire supérieure à 3 heures était associée significativement à un risque plus important de FA (p = 0,04). Après un temps opératoire de 180 min, le risque de FA augmente de 1,3 fois à chaque heure selon Cortina *et al*. [25]. Ceci pourrait être expliqué soit par la contamination péritonéale soit par les perturbation respiratoires et hémodynamiques conséquentes [25]. En effet, dans une étude prospective portant sur 616 malades, Trencheva *et al*.stipulent qu´une durée opératoire importante augmentent le risque de chute des chiffres tensionnels et d´acidose en peropératoire induisant une majoration du risque de FA [26].

Concernant la localisation au niveau de l´angle colique gauche, qui représente une localisation pourvoyeuse de FA pour quelques auteurs [14,27], notre étude a montré également cette relation statistique avec un p= 0,02. Dans notre étude, l´analyse statistique n´a pas trouvé de différence significative entre ces deux groupes (p =0,12). Parmi les limites de notre étude est son caractère rétrospectif qui induit un risque de plusieurs biais. D´une part, les données manquantes et les protocoles opératoires selon les différents opérateurs induisent un biais de sélection des malades. D´autre part, le niveau socio-économique de la population qui consulte notre hôpital représente aussi une source de biais de sélection vu le caractère monocentrique de l´étude.

Il pourrait aussi exister un biais de mesure en rapport avec des erreurs diagnostiques. En effet, nous avons pris en compte la définition large de la FA comme recommandé par la conférence de consensus de 2020 (avis d´experts) [5]. Cependant, toute collection péri anastomotique n´est pas toujours synonyme de FA. Par exemple, un hématome intrapéritonéal surinfecté pourrait avoir les mêmes caractéristiques à l´imagerie. Par ailleurs, notre population n´est pas homogène vue l´inclusion de cancer coliques de différents segments anatomiques et de différentes présentations cliniques, notamment l´inclusion des patients opérés en urgence. Cela pourrait majorer le taux de FA ainsi que le taux de mortalité postopératoire [28]. En effet, vu le nombre limité des patients ayant présenté une FA, l´analyse en sous-groupes était irréalisable. Cela aurait influencé les résultats concernant les facteurs prédictifs de survenue de cette complication.

## Conclusion

Les deux facteurs prédictifs préopératoires indépendants modifiables d´après nos résultats sont l´hypoalbuminémie et l´hémoglobine préopératoire inférieure à 10 g/dl. La prévention de la FA doit s´inscrire dans le cadre d´une prise en charge multimodale du patient avec essentiellement un apport nutritionnel et une correction d´une éventuelle anémie en préopératoire.

### Etat des connaissances sur le sujet

- *La fistule anastomotique reste la complication la plus redoutée après chirurgie colique et continue à poser un problème diagnostique et thérapeutique;*
- *Les facteurs prédictifs de fistule anastomotiques après chirurgie colique sont très fréquemment étudiés conjointement avec ceux du rectum, d´où le risque que les facteurs de risque spécifiques à la chirurgie colique restent dissimulés*.

### Contribution de notre étude à la connaissance

- *Une hémoglobine inférieure à 10 g/dl représente un facteurs prédictif majeur de lâchage anastomotique après chirurgie colique pour cancer; sa correction est essentielle avant toute chirurgie;*
- *La prise en charge multimodale du patient en préopératoire optimise les résultats de la chirurgie colique pour cancer;*
- *La prévention des fistules anastomotiques passe essentiellement par une meilleure sélection des patients candidats à des rétablissements de la continuité digestive lors du même geste opératoire*.
